# A Case of Two Adult Brothers with Wiskott-Aldrich Syndrome, One Treated with Gene Therapy and One with HLA-Identical Hematopoietic Stem Cell Transplantation

**DOI:** 10.1007/s10875-021-01157-6

**Published:** 2021-11-04

**Authors:** Giulia Consiglieri, Francesca Ferrua, San Raffaele Hospital Consortium, Pina Brianti, Jacopo Peccatori, Sarah Markel, Fabio Giglio, Saverio Ladogana, Lucia Dora Notarangelo, Maria Ester Bernardo, Fabio Ciceri, Antonio Bognanni, Maddalena Migliavacca, Federica Barzaghi, Valeria Calbi, Francesca Tucci, Vera Gallo, Francesca Dionisio, Stefania Giannelli, Sabina Cenciarelli, Francesca Ciotti, Federico Fraschetta, Miriam Casiraghi, Giovanni Paolino, Santo Raffaele Mercuri, Alessandro Aiuti, Maria Pia Cicalese

**Affiliations:** 1 Pediatric Immunohematology and Bone Marrow Transplantation Unit, IRCCS San Raffaele Scientific Institute, Milan, Italy; 2 San Raffaele Telethon Institute for Gene Therapy (SR-Tiget), IRCCS San Raffaele Scientific Institute, Milan, Italy; 3 Vita-Salute San Raffaele University, Milan, Italy

To the Editors

Wiskott-Aldrich syndrome (WAS) is a rare X-linked primary immunodeficiency characterized by microthrombocytopenia, recurrent infections, eczema, and increased susceptibility to autoimmunity and tumors. This disease is caused by mutations in the gene encoding for WAS protein (WASp), predominately expressed in hematopoietic cells. WASp alterations can lead to a wide clinical phenotype, ranging from classical to milder forms, depending on the type and severity of the *WAS* gene mutations [1].

The current standard of care is hematopoietic stem cell transplantation (HSCT) from an HLA-identical donor, which is associated with survival rates of 80% or higher in the pediatric population. Particularly, superior 5-year overall survival was observed in patients aged < 5 years at transplant (94%), while the outcome was poorer (66%) in patients > 5 years, due to disease burden. Survival rates were lower in HSCT from mismatched donors, mainly due to transplant-related complications [2–4]. Preliminary data on haploidentical transplants in children are promising [5], although studies are lacking in adult WAS patients.

Over the past decade, ex vivo hematopoietic stem and progenitor cell gene therapy (HSPC-GT) using a lentiviral vector (LV) has resulted in an alternative treatment strategy for WAS patients without a suitable matched donor.

Here we report our experience of two adult siblings with the same *WAS* gene mutation (p.L39P) and similar clinical phenotype, who underwent HSPC-GT and HSCT, respectively.

Our objective is to describe the early and medium-term follow-up after these two different approaches, focusing on safety, clinical outcome, and quality of life (QoL) of the two subjects.

Patient 1 was treated with ex vivo HSPC-GT (OTL-103) under compassionate use at the Pediatric Clinical Research Unit and Pediatric Immunohematology and Bone Marrow Transplantation Unit of the San Raffaele Scientific Institute in Milan. The drug product (autologous CD34 + cells genetically modified with a lentiviral vector encoding for human *WAS* cDNA), trial design, reduced intensity conditioning (RIC), and procedures have been described previously [6] and treatment characteristics are reported in Table 1. The compassionate use program was approved by the independent ethics committee of the San Raffaele Scientific Institute and the Italian regulatory authority (Agenzia Italiana del Farmaco [AIFA]).

**Table 1:** Clinical data at HSCT/GT, transplant characteristics and outcomes, + 3 years after treatment results

**Clinical data at HSCT/GT**		**Patient 1**	**Patient 2**
	**Age of onset/diagnosis of disease**	8	5
	**Clinical manifestations**	Hemorrhagic Diathesis (PLT counts pre splenectomy <10 × 10^9^/L Eczema Autoimmune Thrombocytopenia Lymphadenopathy Vasculitis^**§**^ Herpetic infection Fungal infection	Hemorrhagic Diathesis (PLT counts pre splenectomy <10 × 10^9^/L) Eczema Autoimmune Thrombocytopenia Lymphadenopathy Vasculitis^**§**^ Colliquative adenitis Recurrent infections Pancreatitis
	**Zhu score**	5A	5A
	**Platelets range (× 10** ^**9**^ **/L)**	49-142*	129-154*
	**MPV (fl) (normal value 9.1-12.5)**	9.3	9.9
	**CD3 + (cells/μL)** (normal value:1000-2200) **CD3 + CD4 + (cells/**μL**)** (normal value:530-1300) **CD3 + CD8 + (cells/**μL**)** (normal value:330-920) **CD19 + (cells/**μL**)** (normal value:110-570) **CD16 + CD56 + (cells/**μL**)** (normal value:70-480) **CD4 + CD45 + RA+(cells/**μL**)** (normal value:230-770)	**420** **314** **63** 175 345 155	**641** 539 **88** 209 1152 100
	**Proliferation of T cells in response to mitogens and antigens**	Present to mitogens (below normal limits for aCD3i and PWM), present to candida and tetanus.	Present to mitogens (below normal limits for aCD3i), present to candida and tetanus.
	**% of platelets expressing WASp** **% of lymphocytes expressing WASp (%)**	24.3 9.62	N.D. 1.66
	**Age at treatment (years)**	27	23
**Transplant Characteristics and Outcomes**	**Type of treatment**	HSPC-GT	HSCT
	**Conditioning**	Fludarabine (60 mg/m2 in 2 days) Busulfan (14.7 mg/kg in 2 days). Estimated total Busulfan AUC 48000 ng*h/ml Rituximab (375 mg/m2, 1 dose)	Fludarabine (150 mg/m2 in 5 days) Thiotepa (8 mg/kg in 1 day) Treosulfan (42 mg/m2 in 3 days)
	**Donor type**	Autologous HSPC	MSD (10/10)
	**HSPC source**	PBSC	BM
	**Cell dose (CD34x10** ^**6**^ **/kg)**	16.9 (VCN: 2.3, TE^#^ 97%)	1.9
	**GvHD prophylaxis**	None	Methotrexate (day+1,+3,+6, +11) Cyclosporine from day −1
	**Neutrophil engraftment (days)**	+19	+24
	**Platelet engraftment (days)**	+12	+15
	**Discharge from hospital (days after treatment)**	+26	+36
	**Toxicity related to chemotherapy**	Mucositis grade III	Mucositis grade I
	**Acute GvHD/treatment**	No	Yes (Grade I)/Methylprednisolone and prednisone
	**Chronic GvHD/treatment**	No	Yes/Prednisone, rapamycin, topic tacrolimus, photoapheresis
	**Viral/fungal reactivation**	No	No
	**Severe bleeding events**	No	No
**+ 3 years after treatment**	**Chimerism/engraftment in BM**	VCN/genome: CD34+ 1.33, CD3+ 2.18, CD15+ 1.35, CD19+ 1.81, CD56+ 1.79; LV+ BM CFU 75%	full donor (>97% in peripheral blood and 100% in bone marrow)
	**Platelets (× 10** ^**9**^ **/L) (normal values 130-400)**	227*	308*
	**MPV (fl) (normal value 9.1-12.5)**	9.0	10.5
	**WBC (× 10** ^**9**^ **/L) (normal value 4.8-10.8)**	8.3	6.7
	**Lymphocytes** (normal value: 1400-3300) **CD3 + (cells/μL)** (normal value:1000-2200) **CD3 + CD4 + (cells/μL)** (normal value:530-1300) **CD3 + CD8 + (cells/**μL**)** (normal value:330-920) **CD19 + (cells/**μL**)** (normal value:110-570) **CD16 + CD56 + (cells/**μL**)** (normal value:70-480) **CD4 + CD45 + RA+(cells/**μL**)** (normal value:230-770)	1708 675 **488** **137** 345 504 140	1111 **419** **210** **160** 205 432 N.D.
	**IgG (g/L)** (normal value 8.4-16) **IgA (g/L)** (normal value 0.8-4) **IgM (g/L)** (normal value 0.48-2.2) **IgE (IU/ml)** (normal value 0-100)	7.7 1.6 0.84 4.9	10.93 2.73 0.49 **>5000**
	**Immunoglobulin discontinuation (months after treatment)**	10	24
	**Proliferation of T cells in response to mitogens and antigens**	Normal	Normal
	**Vaccine responses**	Present for HBV^, borderline for tetanus	Absent for HBV and tetanus
	**% of platelets expressing WASp** **% of lymphocytes expressing WASp (%)**	40.4 75.67	74.2 97.2

*N.D.* not done, *HSPC-GT* hematopoietic stem and progenitor cell gene therapy, *HSCT* hematopoietic stem cell transplantation, *VCN* vector copy number, *LV* + *BM CFU* lentivirus positive bone marrow clonogenic progenitors, *PBSC* peripheral blood stem cells, *BM* bone marrow

^*^Splenectomized patients

^§^No active vasculitis at HSCT/GT

^#^ Transduction efficiency

^Positive after a further boost (suggested at + 3-year follow-up)

Patient 2 underwent HSCT at the Unit of Hematology and Bone Marrow Transplantation of San Raffaele Scientific Institute. HSCT was performed from a 10/10 matched sibling donor, using bone marrow as stem cell source, after a myeloablative conditioning (MAC) regimen based on Fludarabine, Thiotepa, and Treosulfan. Detailed transplant characteristics are reported in Table 1. Written informed consents for the procedures were provided by patients.

Patient 1 was the first-born, diagnosed with WAS at the age of 8 years because of thrombocytopenia and bleeding, requiring splenectomy. At the age of 22 years, he showed lower limbs vasculitis with high-titer anti-smooth muscle antibodies. Due to the lack of an HLA-identical donor along with a progressively deteriorating clinical picture, he was treated with ex vivo HSPC-GT (OTL-103) at 27 years of age (Table 1) [6]. He experienced grade 3 mucositis after non-myeloablative conditioning, which required parental nutrition and resolved in 10 days. The HSPC-GT course was otherwise uneventful and resulted in adequate hematopoietic engraftment.

To date, no serious adverse events (SAE) and no oncogenic events have been reported. In terms of efficacy, sustained engraftment of gene-corrected cells resulted in robust WASp expression (displayed in Table 1 and Online resource 1), leading to immune function improvement. No inflammatory or immune-dysregulatory manifestations have been observed. Platelet count normalized, protecting patient from severe-moderate bleeding. Patient’s QoL increased from a score of 88 at baseline to 95 at 3-year post-GT (adult PedsQL).

Patient 2 was diagnosed with WAS in parallel to his older brother (patient 1) and was splenectomized at 5 years. After the onset of vasculitis, which occurred earlier than in the brother, and given the availability of a non-carrier fully-matched sibling donor (MSD), the patient underwent HSCT at 23 years of age (Table 1).

The transplant resulted in a timely hematologic engraftment, full donor chimerism, and adequate immune reconstitution (Table 1). Platelet count normalized and no bleeding events were reported.

However, he developed grade 1 skin acute graft-versus-host disease (aGvHD) during the first month after HSCT, followed by steroid-dependent cutaneous manifestations during the subsequent months initially considered as chronic graft-versus-host disease (cGvHD). As the skin lesions continued despite multiple immunosuppressive treatments, and in consideration of persistent high-titer IgE, a different etiopathogenesis (atopic eczema) was hypothesized. Therefore, dupilumab (anti-IL-4Rα monoclonal antibody inhibiting both IL-4 and IL-13) was started with an induction dose of 600 mg and subsequently 300 mg every 15 days, showing significant clinical benefit (displayed in Online resource 2) and is currently ongoing. Overall, HSCT led to an amelioration of QoL, from a score of 69 to 86 at 3-year post-HSCT, with a subjective worsening in concomitance with the presence of skin lesions.

The management of WAS patients has evolved considerably in recent years. The indication for allogeneic HSCT in WAS was initially restricted to patients with severe manifestations, while other patients were managed conservatively. Nowadays HSCT is considered more broadly and performed in patients with a milder clinical score on the basis of the severity of mutation and the predictive functional WASp impairment. Many adult WAS patients, whose clinical phenotype has evolved during time from less to more severe, would benefit from HSCT, but this is often limited by donor availability and hampered by ongoing infections and/or organ damage. Indeed, despite the great improvement in transplantation procedures, allogeneic HSCT for WAS aged > 5 years is still associated with less favorable outcomes. As a consequence, HSCT is now evaluated individually for each patient [7] and HSPC-GT is emerging as a tailored therapeutic alternative with a potentially more favorable benefit/risk ratio.

Here we report the outcome of HSPC-GT and HSCT in two adult siblings with the same *WAS* mutation. Both treatments resulted in restoration of WASp expression in lymphocytes and platelets, due to sustained engraftment of donor-derived or autologous gene corrected HSPCs. Immune reconstitution was achieved in both patients, with earlier discontinuation of immunoglobulin supplementation in the patient treated with HSPC-GT. Platelet counts, which had raised after splenectomy, further improved and stabilized in normal range, protecting both patients from bleeding events.

In terms of safety, no serious AEs occurred following HSPC-GT. Conversely, the patient treated with HSCT developed GvHD, which triggered chronic and disabling skin signs of immune dysregulation that could be controlled by monoclonal antibody blocking IL-4 and IL-13 signaling, which recently was proved to be beneficial to control severe atopic dermatitis [8]. To our knowledge, this represents the first reported use of dupilumab in WAS. Further studies are required to investigate the pathogenesis of skin manifestations in WAS and the efficacy of different treatments.

In conclusion, the experience from this clinical report in two adult WAS siblings treated with either HSPC-GT or HSCT suggests similar efficacy and safety profile of both therapeutic options, together with maintenance of a good QoL through the entire follow-up. Further to previous reports [9], our case highlights the current availability of an increased range of possible treatment options for adult WAS patients. However, the choice of the most appropriate treatment should be tailored on the single patient, based on donor availability and careful evaluation of patient’s specific risk–benefit profile.

## Supplementary Information

Below is the link to the electronic supplementary material.

Online resource 1 (DOCX 13.9 MB)

Online resource 2 (DOCX 4.28 MB)

## Data Availability

Data captured in the program database or patient notes in addition to all data generated or analyzed during this study are included in this published article.
