# Supplementary material for: A Case of Two Adult Brothers with Wiskott-Aldrich Syndrome, One Treated with Gene Therapy and One with HLA-Identical Hematopoietic Stem Cell Transplantation
Source: J Clin Immunol. 2021 Nov 4;42(2):421–5. doi: 10.1007/s10875-021-01157-6 (PMC8821054; doi:10.1007/s10875-021-01157-6)
Supplement: Supplementary file 1 — Online resource 1 (DOCX 13.9 MB) [file 10875_2021_1157_MOESM1_ESM.docx]

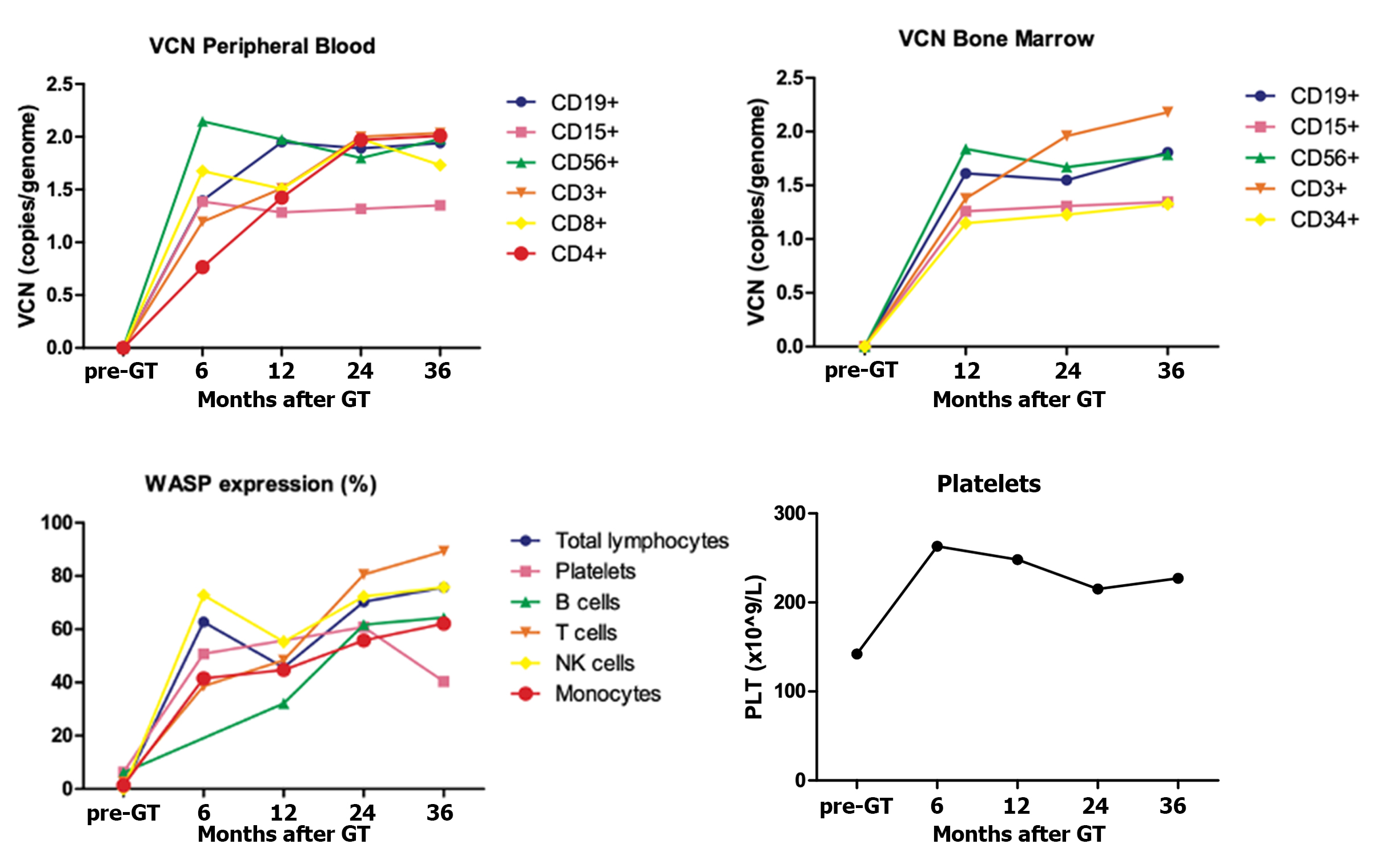


**C**

**D**

**A**

**B**

Online resource 1 was realized with Prism GraphPad

**Online resource 1** Immune reconstitution after GT in Pt1. Engraftment of gene corrected cells expressed as vector copy number (VCN)/genome in sorted subpopulations from peripheral blood (**A**) and bone marrow (**B**), measured by Real Time-PCR after GT. WASP expression (% of WASP+ cells) by flow cytometry in PB cell subpopulations (**C**). Platelet count at different time-points before and after GT (**D**).
