# Supplementary material for: A Case of Two Adult Brothers with Wiskott-Aldrich Syndrome, One Treated with Gene Therapy and One with HLA-Identical Hematopoietic Stem Cell Transplantation
Source: J Clin Immunol. 2021 Nov 4;42(2):421–5. doi: 10.1007/s10875-021-01157-6 (PMC8821054; doi:10.1007/s10875-021-01157-6)
Supplement: Supplementary file 2 — Online resource 2 (DOCX 4.28 MB) [file 10875_2021_1157_MOESM2_ESM.docx]

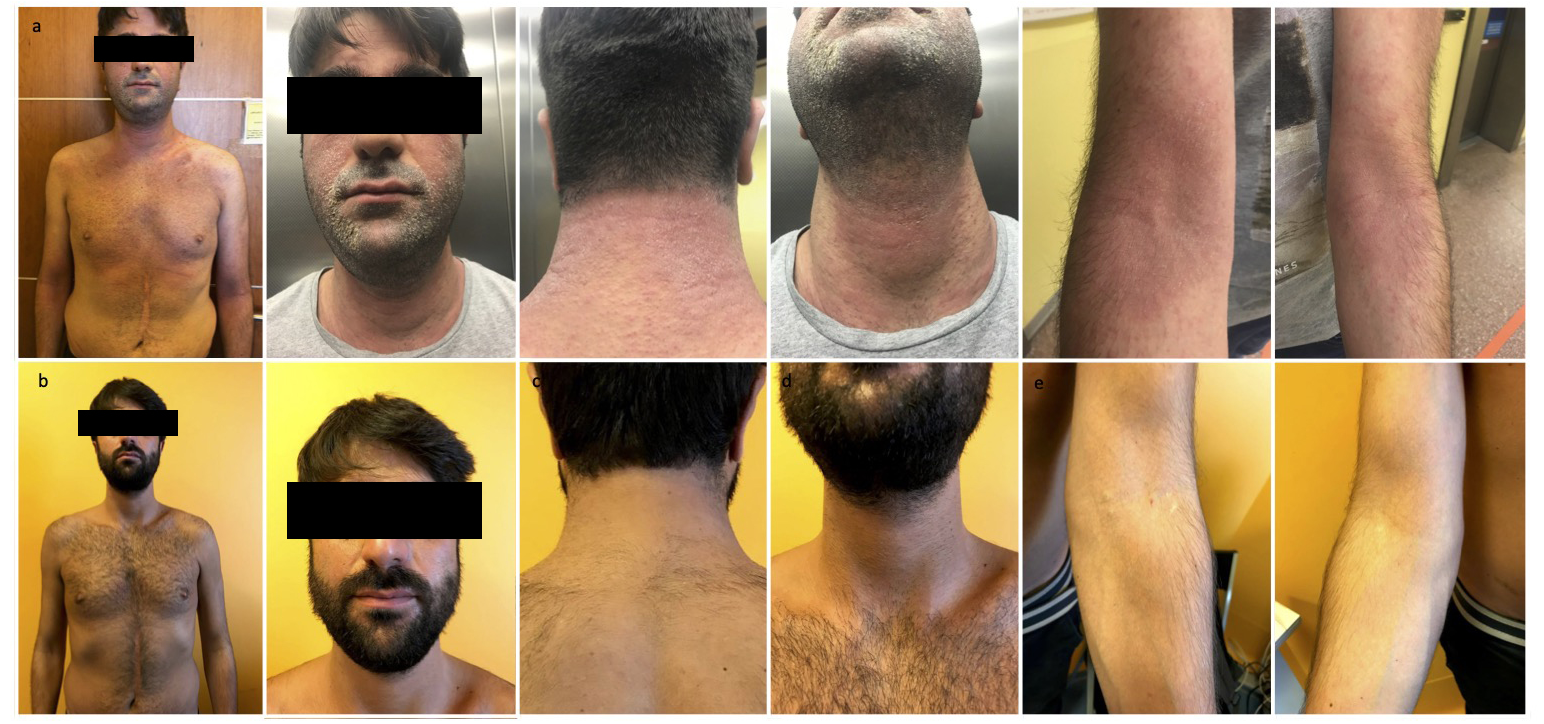


**Online resource 2** Chronic skin manifestations in Pt2 before (**a**) and after dupilumab administration (**b**)
